# Elastoplastic fracture behavior of *Caragana korshinskii* Kom. branches: a discrete element model for biomechanical insights into shrub resource utilization

**DOI:** 10.3389/fpls.2025.1590054

**Published:** 2025-04-29

**Authors:** Qiang Su, Xuejie Ma, Wenhang Liu, Jianchao Zhang, Zhihong Yu, Zhixing Liu

**Affiliations:** College of Mechanical and Electrical Engineering, Inner Mongolia Agricultural University, Hohhot, China

**Keywords:** *Caragana korshinskii* Kom., discrete element flexible model, elastoplastic mechanics, mechanical properties, peak fracture force, GA-BP-GA

## Abstract

**Introduction:**

The interaction between *Caragana korshinskii* Kom. (CKB) branches and crushing machinery is complex, requiring a detailed mechanical model to effectively describe the fracture characteristics of CKB during crushing. This study aims to develop such a model using the discrete element method to simulate the elastoplastic fracture behavior of CKB.

**Methods:**

A mechanical model for CKB was established based on its fracture mechanical characteristics. The model incorporates elastoplastic stages, including elastic, elastoplastic, and fully plastic phases during stem crushing. A parameter calibration method was employed, combining physical experiments with simulation experiments to refine the discrete element model. The key binding parameters of the model were optimized to best simulate the mechanical properties of CKB under various loading conditions.

**Results:**

The optimal binding parameters for the flexible discrete element model were identified as: normal stiffness of 3.67×10^10^ N·m^-3^, shear stiffness of 3.42×10^10^ N·m^-3^, critical normal stress of 6.57×10^8^ Pa, and a binding radius of 0.78 mm. The model successfully replicated the elastic stage force-displacement curve in compression tests with an error of only 0.24%. The discrepancies between simulated and actual fracture forces were 2.79% for compression, 4.68% for bending, 4.14% for shear, and 8.64% for tensile tests, showing good agreement with experimental results.

**Discussion:**

The developed model accurately simulates the elastoplastic fracture behavior of CKB under compression, bending, and shear, providing valuable insights into the crushing mechanism of CKB. The calibration process demonstrated that the proposed DEM model can be an effective tool for exploring and optimizing the crushing process of CKB.

## 1 Introduction

*Caragana korshinskii* Kom. is widely distributed in deserts and saline-alkali areas in the arid and semi-arid regions of northwest China (Su et al., 2024). It serves as the primary tree species for vegetation restoration in desert regions (Zhang and Guo, 2014). To prevent *Caragana korshinskii Kom.* from withering and dying, it necessitates periodic pruning every 3 to 5 years (Wang et al., 2020). Research indicates that *Caragana korshinskii Kom*. branches (CKB) are abundant in crude protein and other nutrients, and once crushed, they can serve as feed for ruminants (You et al., 2022). Nonetheless, CKB possesses a high lignin content and dense structure, rendering them more challenging to crush compared to herbaceous biomass. Thus, exploring the fracture mechanics of CKB and their interaction between CKB and mechanical components holds significant implications for the design of crushing machinery (Chen et al., 2015).

CKB exhibits high hardness and toughness, coupled with certain flexible characteristics. During the crushing process, its stress, deformation, and motion are complex. Traditional experimental research methods (Dong et al., 2023; Miu and Kutzbach, 2008) struggle to accurately analyze the force and motion processes between the machine and the material. In contrast, simulation-based approaches, particularly the discrete element method, effectively overcome this challenge (Zhao et al., 2022). The discrete element method enables detailed analysis of stem deformation, fracture characteristics, and micro-mechanical properties (Guo et al., 2021), demonstrating high precision in simulating the deformation and motion behaviors of plant stems (Xia et al., 2022).

To enhance the analysis the dynamic behavior of stems, lookup tables were constructed based on stem measurements. These measurements were conducted incrementally to assess the impact of plastic deformation (Leblicq et al., 2016). Wang et al. (2020) proposed the elastic hollow cylinder bond model, established a virtual rice plant, and simulated the intricate dehulling process of rice. For simulating the plastic deformation of stems, Liu et al. (2018) developed a flexible wheat straw model based on the Hertz-Mindlin with bonding contact model, capable of simultaneously simulating various deformation behaviors such as bending, tension, and torsion. Zhao et al. (2023) modeled cotton stalks as isotropic structures, established a discrete element model for cotton stalks, and utilized this model to investigate the biomechanical properties of cotton stalks. Although the aforementioned studies can simulate the fracture characteristics of stems to some extent, the modeling process is intricate, and the examination of the fracture mechanics properties of stems is relatively macroscopic, with often neglecting the flexible properties of stems.

For high-density wooden materials, Guo et al. proposed a novel nonlinear elastic-plastic bonded sphere discrete element model to depict the deformation of pine wood. This model can simultaneously account for the irreversible plastic deformation and nonlinear behavior of pine wood (Xia et al., 2019) Guo et al., 2020; (Hamed et al., 2023). Whether investigating the crushing effect of forest wood chips, flowability (Xia et al., 2021), or mechanical structure design (Chen et al., 2022), the discrete element method can significantly enhance research efficiency. However, the aforementioned studies predominantly focus on the secondary crushing of forest wood chips, overlooking the achievement of crushing the entire stem sections. Therefore, constructing a discrete element flexible model is imperative for precisely simulating the behavior of biomass during crushing. Nevertheless, there is currently a dearth of discrete element flexible models for CKB crushing behavior, hindering the intuitive analysis of the deformation, damage, and micromechanical behavior of CKB under the influence of crushers. Consequently, this limitation impedes the multidimensional and in-depth exploration of the intrinsic mechanisms underlying CKB-crusher mechanical interactions. By combining material mechanics theory, a more realistic branch flexibility model was established with the assistance of meta-particles. By comparing numerical simulations with experimental data, the accuracy of the new model in predicting branch deformation and damage, particularly in the fracture behavior of highly flexible branches, is expected to be verified.

Consequently, this research endeavors to elucidate the mechanical evolution principles of CKB subjected to various loading conditions and employs EDEM software to develop a discrete element model of CKB. The model is calibrated through an integrative method that combines both physical experiments and simulation analyses. Initially, intrinsic parameters and contact coefficients of CKB are quantified through physical mechanical testing. This is followed by a detailed analysis of mechanical evolution under different loading conditions. Subsequently, bonding parameters are calibrated via a method integrating compression testing with discrete element modeling, resulting in a CKB discrete element flexibility model with elastic-plastic fracture properties. The optimization of the CKB discrete element flexibility model is performed using response surface methodology and genetic algorithms. Finally, bending and shear tests are conducted to validate the accuracy of the CKB discrete element flexibility model. This study will bridge computational mechanics and plant biophysics by proposing a DEM-based framework that will decode the elastoplastic fracture stages of shrub branches. The insights from this framework will guide for biomechanical optimization in agricultural processing, contributing to more efficient and sustainable practices.

## 2 Materials and methods

### 2.1 Sample preparation

**Figure 1:**
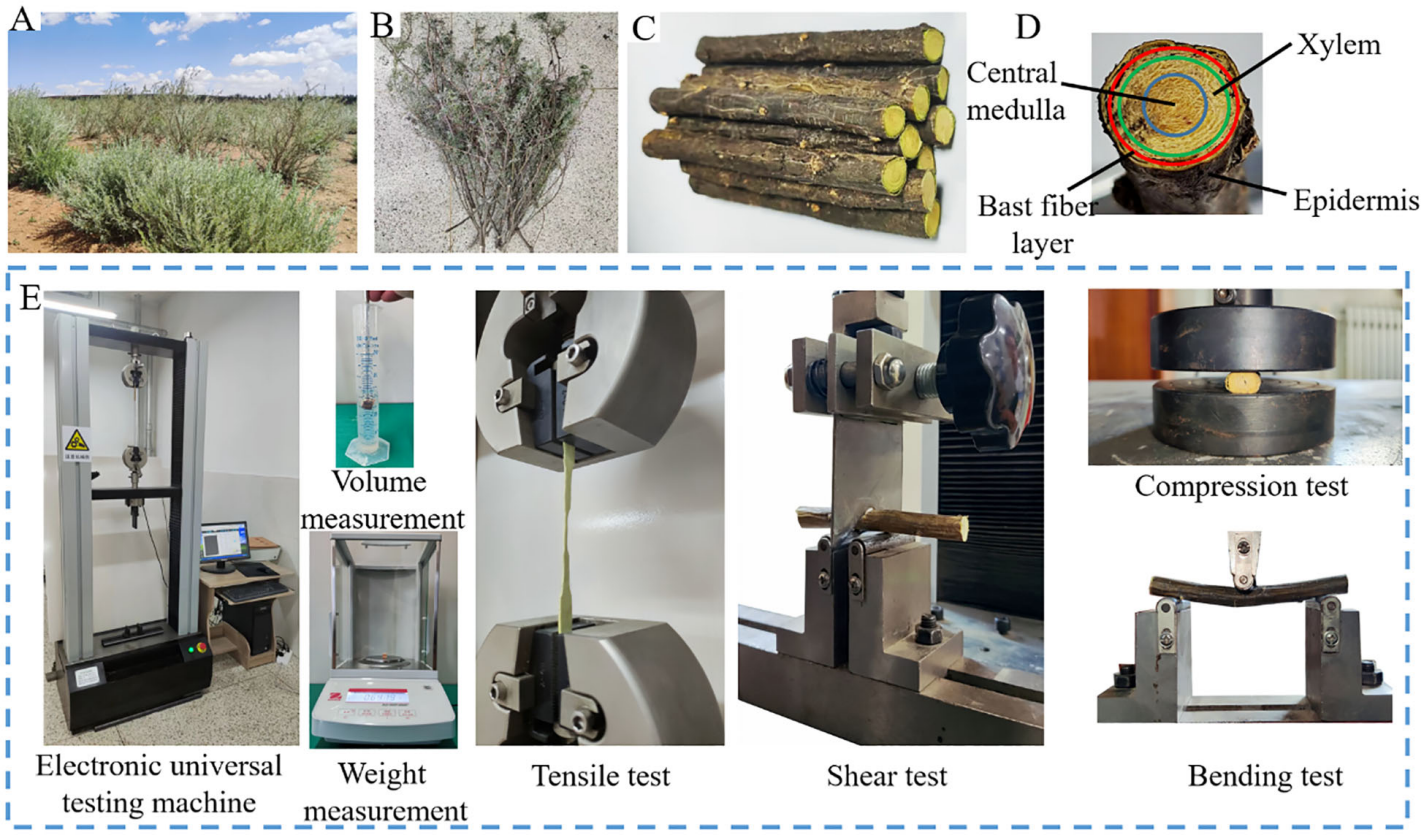
Growth and mechanical tests of CKB. **(A)** Growth status of lemon strips; **(B)** Field sampling; **(C)** Sample; **(D)** Branch components; **(E)** Mechanical testing and equipment.

The CKB samples were collected from the cultivation area in Horinger county in Hohhot City, Inner Mongolia (40°9′36″E, 111°48′N). The variety, *Caragana microphylla Lam.*, were 2–3 years old. As shown in **Figures 1A–C**, the structure of CKB featured a cylindrical cross-section that gradually tapers from the base to the tip. The cross-section consists of the epidermis, cortex, xylem, and pith, arranged from the outermost to the innermost layers (**Figure 1D**). Forty CKB with straight, intact, and robust main stems were randomly chosen, with side branches and small leaves carefully removed. They were encased in plastic film and transported to the laboratory for further investigation. The average diameter of the CKB was measured 10.13 mm at the thick end and 8.87 mm at the thin end. The discrete element model used an average diameter of 9.5 mm.

### 2.2 Measurement of physical property of CKB

#### 2.2.1 Mechanical testing of CKB

Specimens prepared from the primary stems of CKB were designed to withstand tension, compression, bending, and shear loading. Mechanical measurements (including tension, compression, three-point bending, and shearing) were performed in a laboratory setting employing the identical universal testing machine (WDW-30kN, load accuracy ±0.5%, manufactured by Wuhan ShiDai Jinfeng Instrument Co., Ltd.) shown in **Figure 1E**. Different fixtures were used to accommodate various loading modes. The computer recorded the force-displacement alterations of the specimens under different loading modes. Tests were carried out at a loading speed of 10 mm·min^-1^, with five samples subjected to each loading method. The results of the mechanical tests under various loading modes are presented in **Table 1**.

**Table 1:** Results of the mechanical tests.

Item	Values			
	Min	Max	Average	Standard deviation
Tensile force/N	187.69	428.46	312.32	90.44
Compressive force/N	2073.56	1044.4	1553.51	409.02
Bending force/N	258.78	895.60	520.48	162.09
Shear force/N	811.02	2089.94	1376.52	260.47
Elastic modulus/Pa	1.04×10^9^	2.56×10^9^	1.74×10^9^	555.23
Shear modulus/Pa	3.82×10^8^	9.41×10^8^	6.40×10^8^	204.04

#### 2.2.2 Poisson’s ratio *µ* and shear modulus *G*

Tensile testing is commonly used to determine the elastic modulus *E* of plant stems, thereby deriving the shear modulus *G* of the tested material. Throughout the test, CKB samples are secured at both ends using fixtures, and the upper fixture is incrementally raised at a speed of 10 mm·min^-1^ until the sample fractures completely, yielding parameters such as deformation, force, and elastic modulus. The tensile test provides a means to determine of CKB. During elastic deformation, stress is directly proportional to strain. The ratio of stress to strain represents the elastic modulus *E*. The elastic modulus of CKB can be calculated using Equation 1.


(1) $$E=\frac{F/A}{\text{Δ}L/L}$$

where *E* is the elastic modulus (Pa), *F* is the force (N), *L* is the effective length (mm), *A* is the contact area (mm^2^), Δ*L* is the change in effective length (mm).

Given the negligible impact of Poisson’s ratio on the calculation results, the Poisson’s ratio of 0.36 from the Wood Handbook was chosen as the value for CKB.


(2) $$G=\frac{E}{2(1+\mu )}$$

Where *G* is shear modulus(Pa); μ is Poisson’s ratio. The shear modulus of the CKB was found to be 6.40×10^8^ Pa.

The density ρ , Poisson’s ratio μ , shear modulus *G*, and other parameters of CKB are determined based on experimental results. The experimental fixtures are made of steel, and **Table 2** provides the constitutive parameters necessary for constructing the discrete element flexible model.

**Table 2:** Intrinsic parameters of CKB.

Item	Values	
	CKB	Steel
Poisson's ratio	0.36	0.30
Density/kg·m^-3^	984.7	7850
Shear modulus/Pa	6.40×10^8^	7.94×10^10^

#### 2.2.3 Collision recovery coefficient

**Figure 2:**
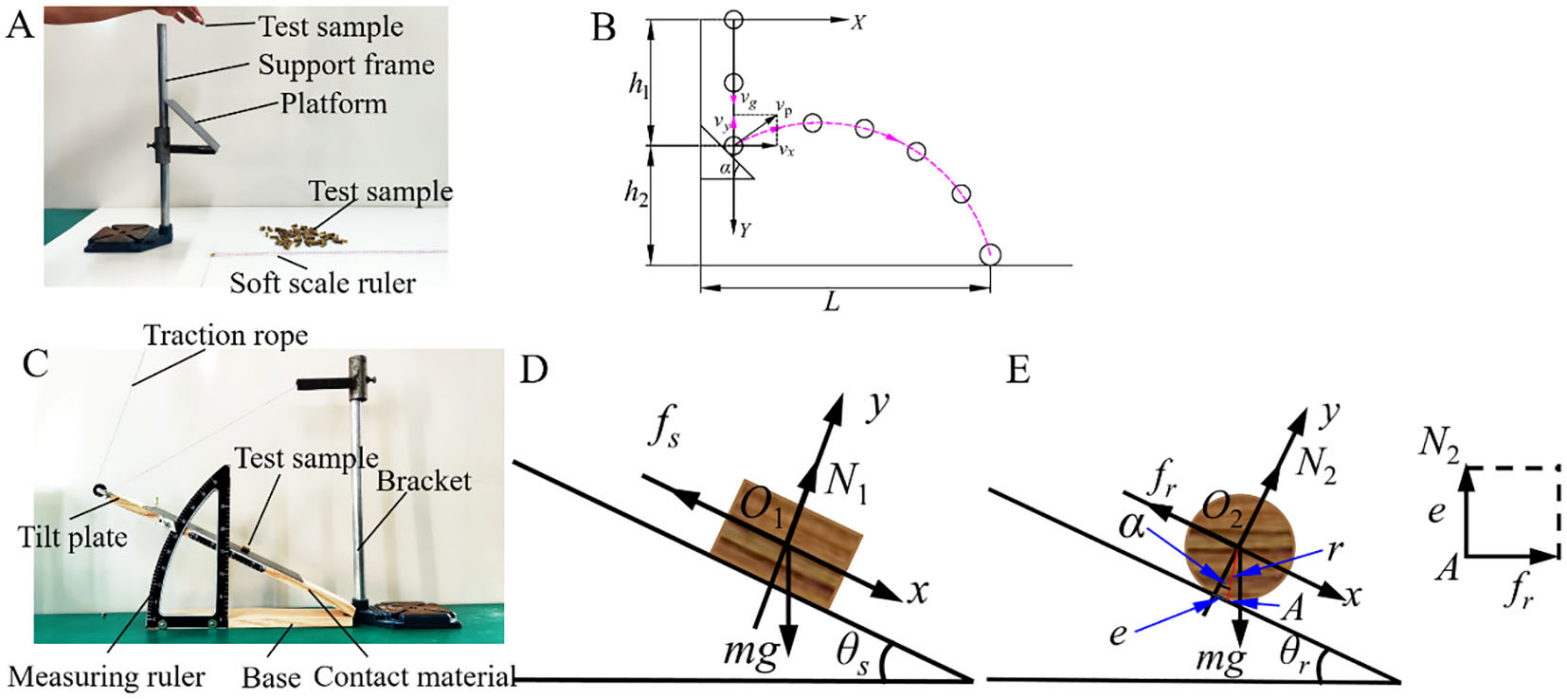
Measurement of collision recovery coefficient and friction coefficient. **(A)** Collision recovery coefficient measurement device; **(B)** Calculation principle of collision recovery coefficient; **(C)** Friction coefficient testing device; **(D)** Principle of static friction; **(E)** Rolling friction principle.

To comprehensively account for the randomness in the initial falling state of the CKB and the effect of its spin during motion, a collision recovery coefficient measurement device was constructed. This device was designed to determine the collision recovery coefficients for “particle-particle” and “particle-geometry” interactions in the discrete element model. Following the methodology outlined by Guan et al. (2022), forty samples of CKB segments were randomly selected for the collision recovery coefficient calibration test. The experimental setup is illustrated in **Figures 2A, B**, with *h*_1_ = 30 mm, *h*_2_ = 40 mm, and *α*=45°. Each test series was repeated ten times, measuring the value of *L*. The resulting collision recovery coefficients ranged from 0.37 to 0.60 for ‘particle-particle’ interactions and from 0.44 to 0.64 for ‘particle-geometry’ interactions

#### 2.2.4 Friction coefficient

The friction coefficient comprises both the static friction coefficient and the rolling friction coefficient. The static friction coefficient represents the ratio of the maximum static friction force exerted on an object to the normal force. The static friction test procedure is shown in **Figure 2C**. In this test, the CKB sample is initially placed axially on a horizontal test board. As the CKB starts to slide, the test board is gradually raised and then halted (**Figure 2D**). At this juncture, record the angle θ indicated on the angle ruler, and compute the static friction coefficient using Equation 3. The static friction coefficient ranges from 0.45 to 0.57 for CKB-CKB interactions and from 0.42 to 0.51 for CKB-steel interactions.


(3) $$\mu_{\text{a}}=\frac{f_{\text{s}}}{N_{1}}=\frac{mg\sin\theta_{\text{s}}}{mg\cos\theta_{\text{s}}}=\tan\theta_{\text{s}}$$

where *μ*_a_ is the coefficient of static friction, *θ*_s_ is the critical angle of static friction (°), *N*_1_ is support force (N), *f*_s_ is friction force (N), m is the mass of the sample (kg), g is gravitational acceleration (m·s^-2^). Rolling friction refers to the resistance encountered when an object rolls over a surface, causing deformation due to the rolling motion. The schematic diagram illustrating the principle of rolling friction is shown in **Figure 2E**. In the experiment, the test plate is positioned horizontally, with the CKB sample arranged radially on its surface. The test board is gradually elevated until the CKB begins to roll, at which point it is halted. The rolling friction coefficient ranges from 0.22 to 0.30 for CKB-CKB interactions and from 0.12 to 0.21 for CKB-steel interactions.

As shown in **Figure 2E**, when the stationary CKB is placed on the inclined plate and begins to roll, with the assumption that the external contact surface deforms, Equation 4 represents the equilibrium forces and couple at point *A*.


(4) $$\left\{ \begin{array}{l} f_{\text{r}}-mg\sin\theta_{\text{r}}=0\\ N_{2}-mg\cos\theta_{\text{r}}=0\\ N_{2}e-f_{\text{r}}\cos\theta_{\text{r}}=0 \end{array}\right.$$

where *f*_r_, *N*_2_ is friction and support force (N), *m* is the mass of the sample (kg), *g* is gravitational acceleration (m·s^-2^), *e* is the vertical distance from point A to axis y (mm), *α* is the angle between *OA* and the y-axis (°), *θ*_r_ is the critical angle of rolling friction (°), *r* is the radius of the CKB (mm).

As the inclined plate is gradually lifted, when the angle between its upper surface and the lower plane of the base reaches the static rolling stability angle, the force equilibrium of the stationary CKB placed on the inclined surface is disrupted, initiating its rolling motion. The condition for rolling is as follows:


(5) $$N_{2}e\leq f_{\text{r}}r\cos\theta_{\text{r}}\to \frac{e}{r}\leq \tan\theta_{\text{r}}•\cos\theta_{\text{r}}$$

In Equation 5, *e*/*r* represents the rolling friction coefficient. Compared to Equation 3, it is evident that the rolling friction coefficient of the material is lower than the sliding friction coefficient. Additionally, the rolling friction coefficient is correlated with the static rolling stability angle and plays a crucial role in enabling CKB to overcome resistance and roll.

### 2.3 Discrete element flexible modelling of CKB

#### 2.3.1 Setting of bonding parameters

**Figure 3:**
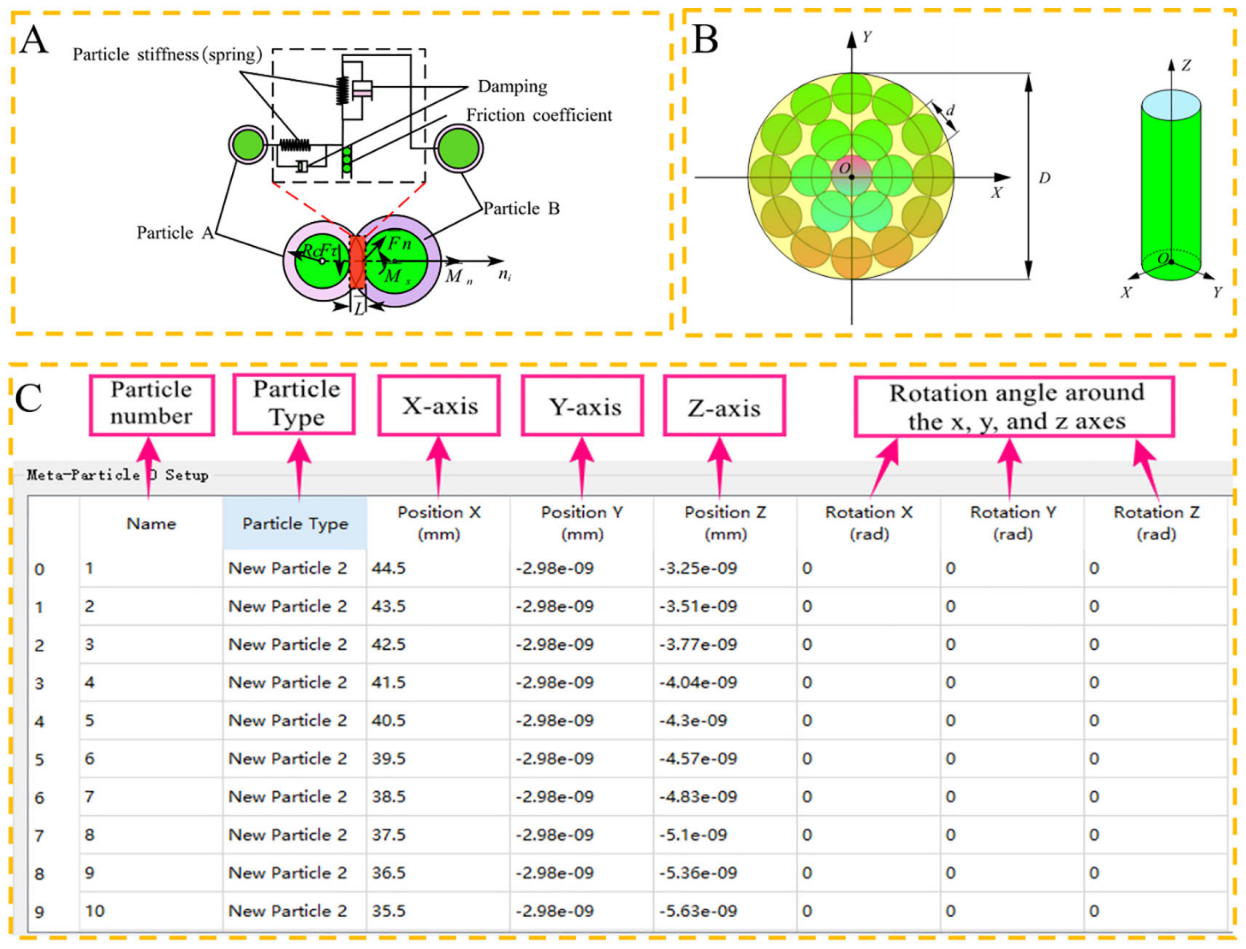
Particle contact model and filling rules. **(A)** Hertz–Mindlin model with the bonding contact; **(B)** Particle generation rules and three-axis spatial coordinates; **(C)** Particle filling rule for discrete element model.

The Hertz-Mindlin with Bonding V2 contact model is used to describe particle-particle interactions. It establishes bonds between adjacent discrete spherical particles that are initially unconnected. This model represents the internal bonding properties of CKB and simulates its evolving behavior under microscopic crushing conditions. As shown in **Figure 3A**, forces can displace particles in both normal and tangential directions.

During the simulation, the forces and torques acting on bonded particles are iteratively updated. The following expressions describe the incremental corrections to the normal and tangential cohesive forces and moments over a single time step *δ*_t_, which are accumulated over time to compute the total bonded interactions As demonstrated in Equations 6, 7:


(6) $$\left\{ \begin{array}{l} \delta F_{n}=\nu_{n}K_{\text{n}}A_{\text{b}}\delta_{t}\\ \delta F_{t}=-\nu_{t}K_{\text{s}}A_{\text{b}}\delta_{t}\\ \delta M_{n}=-\omega_{n}K_{\text{s}}J\delta_{t}\\ \delta M_{s}=-\frac{1}{2}\omega_{t}K_{\text{n}}J\delta_{t} \end{array}\right.$$

where


(7) $$\begin{array}{l} A_{b}=\text{π}\cdot R_{ab}^{2}\\ J=\frac{1}{2}\text{π}\cdot R_{ab}^{4} \end{array}$$

where *δF*_n_ and *δF*_s_ represent the normal and tangential adhesion forces of the bonding key, *δM*_n_ and *δM*_s_ represent the torques of the bonding key in the normal and tangential. *δ*_t_ is time step in the simulation (s), $v_{n}$ , $v_{\text{t}}$ , $\omega_{n}$ , $\omega_{\text{t}}$ are the normal and tangential velocities, and the normal and tangential angular velocities, respectively. $K_{n}$ , $K_{s}$ are, respectively, the shear and normal bond stiffness, $A_{\text{b}}$ and J are, respectively, the moment of inertia, the polar moment of inertia, and the cross-sectional area of the bond. $R_{ab}$ is the bond radius, which depends on the diameter of the smallest sphere in contact.

When the normal and tangential stresses ( $\sigma_{\max},\tau_{\text{max}}$ ) between two adjacent particles attain their maximum values, bond rupture occurs (Potyondy and Cundall, 2004; Liu et al., 2019). Consequently, the fracture behavior of CKB can be simulated by modeling its bonding state. The mathematical expressions for the normal and tangential ultimate stresses are presented in Equations 8 and 9.


(8) $$\sigma_{\max}<\frac{-F_{n}}{A_{\text{b}}}+\frac{2M_{\text{s}}}{J}R$$

(9) $$\tau_{\max}<\frac{-F_{\tau }}{A_{\text{b}}}+\frac{M_{n}}{J}R$$

where $\sigma_{\max}$ is the normal ultimate stress (Pa), $\tau_{\max}$ is the tangential ultimate stress (Pa), $A_{\text{b}}$ is the contact area.

The configuration of particle-particle bonding parameters is critical for the successful construction of the model. If the parameter is too small, the bond stiffness is insufficient to withstand the external forces compared to the external forces acting on the particles, resulting in unsuccessful bonding between neighboring particles and model “collapse”. Conversely, if the parameter is too large, the bond stiffness exceeds the critical load-bearing capacity, causing excessive elastic deformation in the particle contact areas, which may result in particle bouncing and potential “explosions.” Significant differences exist in the parameter combinations of bonding bonds among different discrete element models (Hu et al., 2023; Li et al., 2024).

#### 2.3.2 A discrete element modeling methodology for CKB

A rapid stem modelling approach is proposed to integrate the concept of discrete element particle bonding. The core methodology involves importing the 3D coordinates of the center of mass of all spherical particles into EDEM software, followed by utilizing the Hertz-Mindlin with bonding contact model to “bond” all the particles into a “polymer”. Subsequently, the Hertz-Mindlin contact model is applied to “bond” all particles into an “aggregate”. To improve simulation accuracy, the molded model is designed to closely resemble the biological characteristics of the crop while also being simplified to improve simulation efficiency. The cross-section of CKBs to a circle transforms the particle-filling challenge of the discrete element model into a problem of “filling a large circle with a small circle”. Filling circles in bounded shapes has been an intriguing problem for a long in pure mathematics literature (Zadorozhnyi et al., 2024).

**Figure 3B** illustrates the particle generation process for a circular cross-section. In this depiction, the yellow circular region represents the particle template to be filled, and the circle is partitioned into a grid. The initial particle is generated at the center of the contour, and subsequent particles are generated around the contour according to the set particle-particle contact radius, extending outward to a radius *R*_ab_ before generating additional particles. This iterative process continues until the entire region is filled, forming a discrete element model. As particles accumulate layer by layer along the length direction of the stem, the CKB discrete element model is ultimately established.

The microstructural characteristics of CKB are characterized by the concentric annual rings surrounding the pith core, a consequence of the wood’s incremental growth layers during each growth period. As CKB undergoes prolonged growth, its xylem becomes hardened, the pith core thins, and the epidermis hardens through natural air drying (Wang et al., 2020), significantly impacting the cutter’s performance. To enhance computational efficiency, the model was simplified. Additionally, CKBs were treated as isotropic due to their radial loading during cutting. The identical particles were employed to represent the epidermis, xylem, and pith of CKB.

The three-axis spatial coordinate method was employed to precisely construct the desired physical structure model with a minimal number of particles, ensuring both computational efficiency and model fidelity. The CKB was approximated as a cylinder, *representing* the stalk’s cross-section in the spatial coordinate system *OXYZ*. The *Z*-axis signifies the stalk’s axial direction, with point *O*(0, 0, 0) as the cross-section center coordinate. Particle position is determined by its three-axis coordinates (*X*, *Y*, and *Z*), while cross-section particles are arranged in a circular pattern resembling a tree’s shape. All constructed meta particle cross sections were stacked along the axial direction without gaps to form the desired length discrete metamodel.

The 3D model can be generated using 3D modeling software. The specific operational steps are outlined below:

a. Export the 3D model representing the actual shape and size of the CKB to a.x_t file format. Utilizing the three-axis spatial coordinate method, Hypermesh 2020 performs meshing with a mesh size set at 1 mm, and subsequently, the meshed model is saved in.msh format.
b. Hypermesh 2020 software calculates essential data, including the spatial 3D coordinates of each node in the mesh model, the number and names of nodes, and the actual mesh radius. The delineated mesh’s center point coordinates serve as the meta-particle coordinates derived from the mesh coordinates.
c. Particle creation rules are devised based on the template shown in **Figure 3C**, specifying parameters such as particle number, type, three-axis coordinates, and rotation angles along each axis. Within the EDEM software, a new “meta-particle” sub-file is established, and upon importing the creation rules, a singular CKB discrete meta-simulation model can be generated. This model serves as a particle template for subsequent simulations.

**Figure 4:**
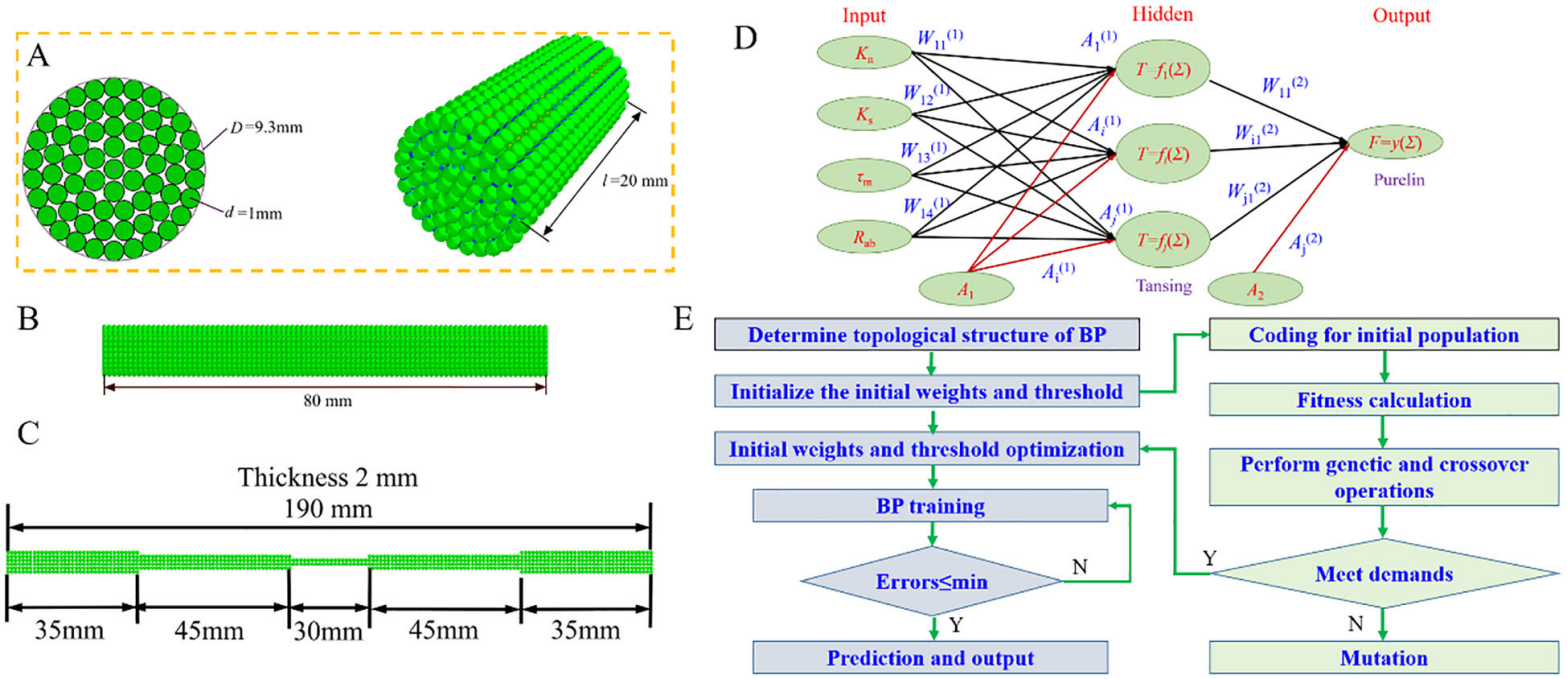
Discrete element model and parameter optimization method. **(A)** Compression model; **(B)** Bending and shear model; **(C)** Stretch model; **(D)** A general schematic of a three-layer neural network; **(E)** Flow chart of combined GA-BP optimization.

In this investigation, the parameter setting for “Hertz-Mindlin with Bonding V2” adopts empirical values to ensure firm bonding between each particle forming the discrete metamodel of the CKB, thereby avoiding any “collapse” or “explosion”. The CKB growth area feature diameters uneven terrain, with the most statistically obtained cutting locations situated 20~30 cm above the ground. Consequently, the diameters of this area were measured, ranging from 8.87 to 10.13 mm, with an average diameter of 9.50 mm considered for the discrete element model. However, due to the inability to closely tangent particle diameters, the diameter of the CKB discrete element model was adjusted to 9.30 mm. **Figures 4A–C** illustrates the CKB discrete element flexibility model utilized for the calibration tests.

Within the Hertz-Mindlin model, the interaction force between two discrete CKB element model particles is governed by parameters such as the standard stiffness per unit area, shear stiffness per unit area, critical normal stress, critical shear stress, and bonding radius. This study draws upon analogous crop stems to initially establish the parameter ranges for the standard stiffness per unit area, shear stiffness per unit area, critical everyday stress, and critical shear stress of the CKB discrete element model (Liu et al., 2023), as illustrated in **Table 3**.

**Table 3:** The levels of Plackett-Burman test for particle.

Parameter	Code name	Parameter coding	
		Low (-1)	High (+1)
Particle-particle collision recovery coefficient	*A*	0.37	0.60
Particle-particle coefficient of static friction	*B*	0.45	0.57
Particle-particle coefficient of kinetic friction	*C*	0.22	0.3
Particle-geometry collision recovery coefficient	*D*	0.44	0.64
Particle-geometry's static friction coefficient	*E*	0.42	0.51
Particle-geometry's coefficient of kinetic friction	*F*	0.12	0.21
Particle-particle normal stiffness per unit area/N·m^-3^	*K*_n_	1×10^9^	1×10^11^
Particle-particle shear stiffness per unit area/N·m^-3^	*K*_s_	1×10^9^	1×10^11^
Particle-particle critical normal stress/Pa	*σ*_m_	1×10^7^	1×10^10^
Particle-particle critical shear stress/Pa	*τ*_m_	1×10^7^	1×10^10^
Bonding radius/mm	*R*_ab_	0.6	1

### 2.4 Parameter calibration method

Parameters such as particle radius, Poisson’s ratio, and elastic modulus in the CKB discrete element model are directly related to the particle-particle bonding parameters, which serve as the primary initial values for simulations in EDEM. Therefore, calibrating the simulation parameters of the CKB discrete element model is crucial for improving its accuracy. Compression tests were conducted to calibrate parameters related to “particle-particle” and “particle-geometry” interactions, with the focus on determining the peak damage force during the CKB compression simulation.

A Plackett-Burman test was performed using Minitab 2019 software to identify the factors with significant effects. The peak destructive force was used as the response variable, while the simulation parameters for contact were defined as shown in **Table 3** (Chen et al., 2025). The setup simulation test comprised eleven real parameters, each set to low level (-1) and high level (1) based on recommended range values. The simulation trials included one center point and a total of twenty-one sets, each repeated three times to calculate the average damage resistance, denoted as *F*_max_.

Based on the results of the Plackett-Burman test for selecting significant parameters, a steepest ascent test was designed to narrow the parameter range. In the simulation test, non-significant parameters were set to the midpoint of their range, while the specified increments gradually increased significant parameters.

A Central Composite Design test was implemented using response surface methodology, following the results from the steepest ascent test. Significant parameters were set at high, medium, and low levels, coded as 1, 0, and -1, respectively, while non-significant parameters were kept constant, based on the steepest ascent test results. Five centroids were used to estimate the error. A total of twenty-seven test sets were conducted, each repeated three times, and the mean values were recorded as the numerical results for each set.

### 2.5 Optimal extraction condition by GA-BP-GA

Due to the complex interplay between independent and dependent variables, MATLAB was employed to perform GA-BP regression fitting modeling (Parida et al., 2024) on the data acquired through central composite design, ensuring the consistency between the predicted values from the quadratic polynomial model and experimental data and extracting optimal bonding parameters. To mitigate over-training and over-parameterization risks, the entire dataset (comprising 81 groups) was randomly partitioned into 57 groups (70%) for training and 24 groups (30%) for testing (Xu et al., 2022). Furthermore, to expedite neural network gradient descent while enhancing accuracy in finding the optimal solution, the mapminmax function was employed for input and output data normalization (Ma et al., 2023).

The BP neural network (BPNN) is a classic back-propagation neural network, shown in **Figure 4D**. Comprising three layers—input layer, hidden layer, and output layer—it integrates external input data processed by the “tansig” activation function in the hidden layer and the “purelin” output function in the output layer. Subsequently, the output layer neurons deliver results (Xu et al., 2022). The training target error is set at 0.001, with a learning rate of 0.001 and a maximum of 200 training steps. The input layer consists of four neurons: normal contact stiffness (*K*_n_), tangential contact stiffness (*K*_s_), critical tangential stress ( $\tau_{\text{m}}$ ), and contact radius (*R*_ab_), with the maximum compression force *F*_max_ designated as the output layer. The number S of hidden layer nodes is determined through trial and error. The formula for calculating the number S of hidden layer nodes is:


(10) $$S=\sqrt{n+l}+c$$

where *n* and *l* are the number of neurons in the input layer and output layer, *c* is a constant, ranging from 1 to 10 (Qi et al., 2019), the values of *S* calculated from Equation 10 range from 4 to 13.

Before executing the BP neural network, a genetic algorithm is used to optimize the initial weights and thresholds of the hidden and output layers, addressing issues such as local minima. The genetic algorithm iteratively improves the population of individuals through selection, crossover, and mutation. Once optimized, the initial weights and thresholds derived from the genetic algorithm are applied to the BP neural network, which then learns and updates the model until the termination criterion is met (Salim et al., 2019), as shown in **Figure 4E**. The genetic algorithm parameters are configured as follows: 200 evolutionary iterations, a population size of 200, the norm Geom Select selection function with a coefficient of 0.09, a crossover coefficient of 0.8, and a mutation coefficient of 0.2 (Gammoudi et al., 2021).

Optimizing unknown nonlinear functions purely based on input-output data is challenging. To address this, the genetic algorithm’s nonlinear optimization capabilities are utilized, with the neural network model acting as the fitness function for the optimization process. The goal of the optimization is to achieve a maximum compression force of 1553.51 N while simultaneously optimizing four bonding parameters. The genetic algorithm is configured with 100 evolutionary iterations, a population size of 200, the normGeomSelect selection function with a coefficient of 0.8, a crossover coefficient of 2, and a mutation coefficient of 0.2.

### 2.6 Bending, shear and tensile test verification

Building upon the aforementioned research, the optimal combination of CKB bonding parameters was determined. To delve deeper into the efficacy of these parameters in delineating the bending and shear mechanical properties of CKB, a CKB discrete element flexibility model measuring 9.3 mm in diameter and 90 mm in length was developed. **Figure 4A** depicts a three-point bending test, while **Figure 4B** illustrates a double-support cutting test. The tool thickness is set 1.5 mm, with an included angle of 150°, and a loading speed of 10 mm·min^-1^ is applied. **Figure 4C** shows the tensile test.

## 3 Results and discussion

### 3.1 Mechanical analysis of elastic plastic crushing of CKB

**Figure 5:**
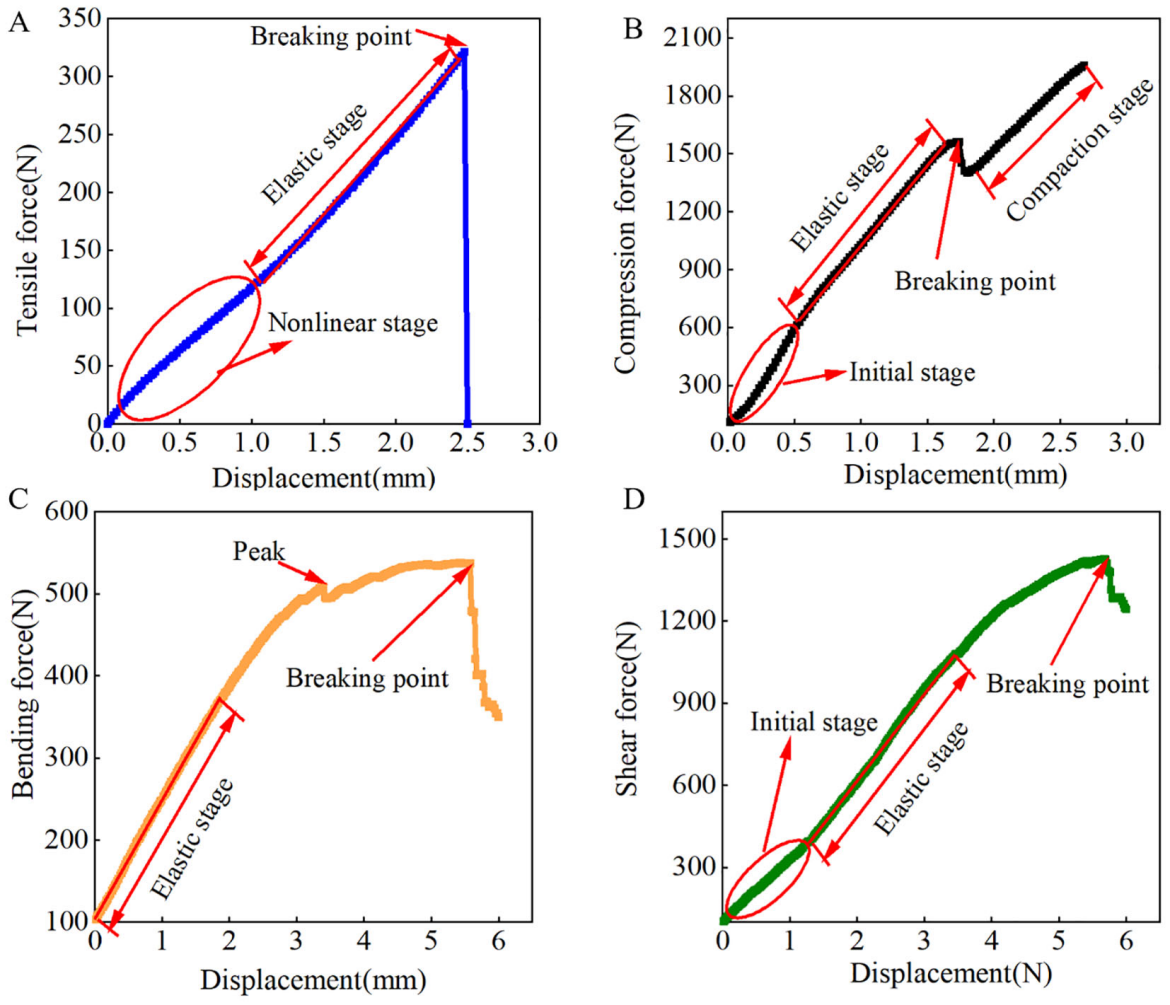
Force versus displacement for different loading modes. **(A)** Tensile test; **(B)** Compression test; **(C)** Bending test; **(D)** Shear testl.

The mechanical relationship of CKB sample tensile failure are illustrated in **Figure 5A**. The curve exhibits two distinct stages: a nonlinear phase followed by a linear elastic phase. In the initial phase of the tensile test, the force increases gradually as the displacement rises, which can be attributed to sliding between the clamp and the specimen. Subsequently, the force increases linearly with displacement, accompanied by ongoing deformation until the tensile force reaches its peak. Upon exceeding the ultimate strength, the specimen undergoes a sudden fracture, characterized by a sharp drop in force without a discernible plastic zone— a behavior typical of brittle materials.

**Figure 5B** depicts the CKB’s resistance to compression deformation. The compression process progresses through distinct stages: elastic, plastic, and compaction. Initially, the softness of the skin leads to a nonlinear increase in force, followed by linear elastic deformation of the CKB. With continued loading, if the maximum compression force exceeds the crushing strength of the CKB, it fractures and undergoes permanent plastic deformation. Further deformation results in a significant increase in compression, leading to gradual compaction of the CKB under sustained compressive force.

**Figure 5C** illustrates CKB’s resistance to bending deformation. Initially, the bending force increases with displacement in the elastic stage, transitioning into the viscoelastic failure stage. During this phase, the bending force increases gradually alongside increased deformation. Upon reaching the peak value due to the indenter, rupture of the outer skin occurs at this location, resulting in a sudden drop in load followed by subsequent plastic deformation.

**Figure 5D** shows the force-displacement curve observed during the shear test. Initially, the curve exhibits a nonlinear growth stage, with the shear force increasing as displacement rises. Subsequently, as displacement continues to increase, the shear force undergoes linear elastic changes. Similar to the bending test, during the viscoelastic stage, the shear force gradually increases, culminating in the maximum load value. Ultimately, CKB failure occurs, accompanied by a rapid decrease in load as displacement increases, marking the end of the curve upon complete sample detachment.

### 3.2 Test results of parameter calibration process

#### 3.2.1 Plackett–Burman design test analysis

The significance of each factor is determined using the Plackett–Burman design test, which compares the difference between the two levels of each factor with the overall difference, enabling efficient identification of factors that significantly influence the response value (Spadi et al., 2021). Design-Expert 13 software is used in this study, with *F*_max_ serving as the response variable, to identify factors significantly impacting *F*_max_. For each factor, high and low levels are selected, a center point is selected, and a total of 21 tests are conducted, as shown in **Table 4**.

**Table 4:** Design and results of Plackett-Burman test scheme.

NO.	A	B	C	D	E	F	*K*_n_	*K*_s_	*σ_m_*	*τ_m_*	*R_ab_*	*F*_max_
1	1	1	1	-1	-1	1	1	-1	1	1	-1	1097.85
2	1	-1	1	1	-1	-1	-1	-1	1	-1	1	149.85
3	-1	-1	-1	1	-1	1	-1	1	1	1	1	1167.05
4	1	1	-1	-1	1	1	-1	1	1	-1	-1	6.19
5	1	-1	-1	-1	-1	1	-1	1	-1	1	1	1612.92
6	-1	1	1	-1	1	1	-1	-1	-1	-1	1	155.47
7	-1	-1	1	1	-1	1	1	-1	-1	-1	-1	443
8	1	1	-1	-1	-1	-1	1	-1	1	-1	1	515.08
9	0	0	0	0	0	0	0	0	0	0	0	849.58
10	1	-1	1	-1	1	1	1	1	-1	-1	1	716.83
11	-1	1	1	-1	-1	-1	-1	1	-1	1	-1	7.11
12	1	-1	-1	1	1	-1	1	1	-1	-1	-1	625.17
13	-1	1	-1	1	-1	1	1	1	1	-1	-1	724.18
14	1	1	1	1	-1	-1	1	1	-1	1	1	1694.48
15	-1	-1	-1	-1	-1	-1	-1	-1	-1	-1	-1	1.92
16	1	-1	1	1	1	1	-1	-1	1	1	-1	84.01
17	-1	-1	1	-1	1	-1	1	1	1	1	-1	2610.67
18	1	1	-1	1	1	-1	-1	-1	-1	1	-1	22.99
19	-1	-1	-1	-1	1	-1	1	-1	1	1	1	1356.97
20	-1	1	-1	1	1	1	1	-1	-1	1	1	1022.18
21	-1	1	1	1	1	-1	-1	1	1	-1	1	1021.29

**Figure 6:**
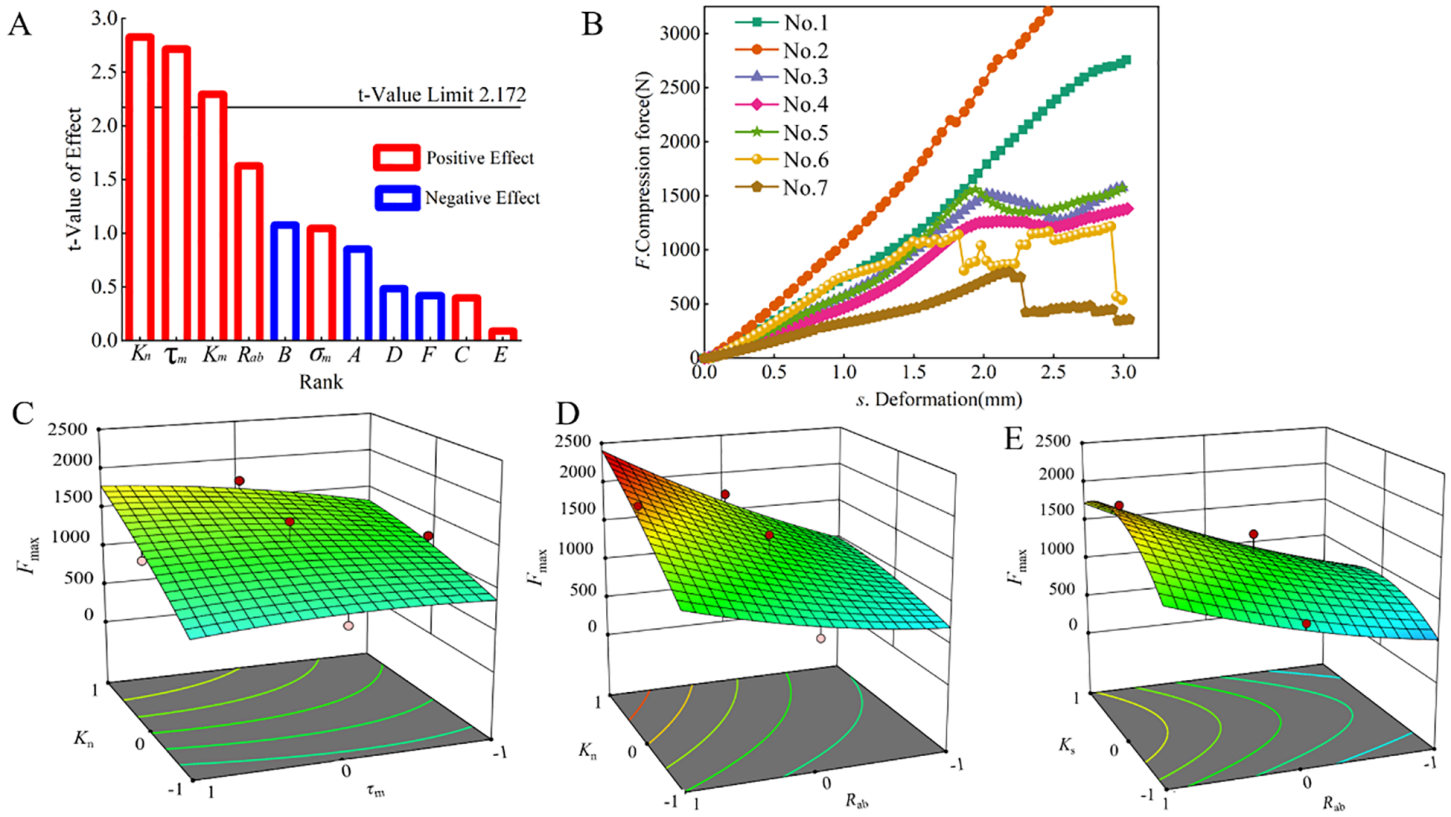
Pareto chart and response surface graph. **(A)** Pareto chart; **(B)** Effect of bonding model parameters on *F*_max_; **(C-E)** Response surface of the optimal solution of parameter calibration.

**Figure 6A** depicts the Pareto diagram illustrating the results of the Plackett–Burman design test. The factors affecting *F*_max_ from large to small are: *K*_n_, *τ*_m_, *K*s, *R*_ab_, *B*, *σ*_m_, *A*, *D*, *F*, *C*, *E*, *K*_n_, *τ*_m_ and *K*_s_ are identified as the significant influencing factors positively correlated with *F*_max_. Overall, bonding parameters exert a more substantial impact on *F*_max_, thus warranting the test’s focus on them.

#### 3.2.2 Steepest climb test analysis

The steepest climb test can be used to study the impact of factor levels on evaluation indicators and further narrow the range of factor levels. Research has found that contact parameters have a small impact on *F*_max_ (Bryan et al., 2021). The contact parameters have been measured through experiments in the Materials and methods section. In subsequent experiments, the average is selected for simulation calculations, and the impact of the contact parameters on the maximum compression force is no longer considered. Based on the results from the Plackett–Burman design test, this study focused solely on conducting the steepest ascent test for the bonding parameters to investigate their influence on the force-deformation curve. The relative error Δ*E* is given by Equation 11.


(11) $$\Delta E=\frac{|F_{\max}-F_{\text{ave}}|}{F_{\text{ave}}}\times 100$$

where *F*_ave_ is the average value of *F*_max_ (N).

Following iterative experimental adjustments, it was noted that the experimental design outcomes shown in **Table 5** closely resemble the actual values. It is worth noting that the relative errors for tests No. 3 and No. 5 were the smallest, at 6.93% and 6.02% respectively. Building upon these findings, subsequent central composite design test were executed.

**Table 5:** Steepest climb test method and results for bonding parameters.

No.	*K*_n_	*K*_s_	*τ_m_*	*F*_max_	Δ*E*(%)
1	5×10^10^	5×10^10^	1×10^10^	2785.90	79.33
2	5×10^10^	3×10^10^	1×10^9^	2296.34	47.82
3	5×10^10^	1×10^10^	1×10^8^	1445.99	6.93
4	3×10^10^	1×10^10^	1×10^10^	1269.11	18.31
5	3×10^10^	3×10^10^	1×10^9^	1459.89	6.02
6	3×10^10^	5×10^10^	1×10^8^	1100.32	29.17
7	1×10^10^	1×10^10^	1×10^10^	933.54	39.91

Values in parentheses represent *ΔE*. The other parameters are as follows: the contact parameters are all taken to the 0 level, *σ*=5.005×10^9^ Pa, and *R_ab_* =0.8 mm.

**Figure 6B** shows the deformation curve obtained from the climbing test. The variations during the elastic stage across all curves are largely consistent, displaying a trend of linear growth. Higher values of *K*_n_, *K*_s_, and *R*_ab_ are associated with increased compressive strength for P1 and P2, with the maximum compression force exceeding 2000 N. Curves corresponding to tests P3 to P7 exhibit a similar trend in compression force the evolution, characterized by an initial increase followed by a decrease. At an average deformation of 2.02 mm, the yield point is attained, leading to a gradual decrease in compressive strength. This is followed by a stage of compression and densification, during which compressive strength gradually increases. Consequently, *K*_n_, *K*_s_ and *R*_ab_ not only affect *F*_max_, but also significantly influence the *F*-*s* curve trend. Therefore, the parameter range of *K*_n_ and *K*_s_ is further determined to be 1×10^10^ to 5×10^10^ N·m^-3^.

#### 3.2.3 Response surface test analysis

To determine the optimal combination of bonding parameters, a four-factor, three-level central composite experimental design was executed utilizing Design-Expert 13 software. Based on the findings from the Plackett-Burman design and steepest climb tests, the contact parameters were set to the 0 level. A total of 27 tests were conducted, with the average of three repeated tests serving as the test outcome. **Table 6** illustrates the experimental design and corresponding results.

**Table 6:** Central composite design test and results.

No.	*K*_n_	*K*_s_	*τ*_m_	*R*_ab_	*F*_max_
1	1	1	1	-1	706.67
2	1	-1	-1	-1	198.75
3	1	-1	1	-1	844.46
4	0	0	0	-1	718.49
5	-1	-1	-1	1	599.05
6	1	-1	-1	1	1271.36
7	1	1	-1	-1	486.46
8	1	-1	1	1	2113.33
9	-1	-1	1	-1	202.48
10	0	0	1	0	1237.14
11	0	0	-1	0	1236.68
12	1	1	1	1	2273.51
13	-1	1	1	-1	264.38
14	0	0	0	0	1562.00
15	-1	-1	1	1	602.16
16	-1	1	1	1	1037.15
17	0	0	0	1	2050.50
18	0	0	0	0	1236.68
19	0	1	0	0	919.73
20	-1	1	-1	-1	248.21
21	-1	1	-1	1	1026.42
22	1	1	-1	1	2051.28
23	-1	-1	-1	-1	450.22
24	-1	0	0	0	760.33
25	0	0	0	0	1258.68
26	1	0	0	0	1722.05
27	0	-1	0	0	915.53

After analyzing the results of the central composite design test, a second-order regression model was developed to explain the factors influencing CKB under compressive force, as shown in Equation 12.


(12) $$\begin{array}{c} F_{\max}=1317.76+359.86K_{n}+100.91K_{s}+95.16\tau_{m}+494.7R_{ab}+22.99K_{n}K_{s}\\ +134.24K_{n}\tau_{m}+210.85K_{n}R_{ab}-48.36K_{s}\tau_{m}+112.04K_{s}R_{ab}\\ +27.73\tau_{m}R_{ab}-59.23K_{n}{\;}^{2}-382.74K_{s}{\;}^{2}-63.51\tau_{m}{\;}^{2}+84.04R_{ab}{\;}^{2} \end{array}$$

**Table 7** presents the variance analysis results of the quadratic regression model. The regression model (*p*<0.001) indicates a highly significant relationship between the maximum compression force and the derived regression equation. The lack of fit term (*p*=0.6584>0.05) suggests a small proportion of abnormal errors between the derived regression equation and the actual fitting, indicating a good fit. With a coefficient of determination (*R*²=0.9652) and an adjusted determination coefficient (*R*_adj_²=0.9247), along with an accuracy of 16.05%, the regression model exhibits satisfactory accuracy. Additionally, *K*_n_, *R*_ab_, *K*_n_ *τ*_m_, *K*_n_ *R*_ab_, and *K*_s_² demonstrate extremely significant effects on *F*_max_ (P<0.01). *K*_s_, *τ*_m_ and *K*_s_ *R*_ab_ exhibit significant effects on *F*_max_ (*p*<0.05), while the remaining factors are not significant. The order of influence of the four parameters on *F*_max_ is *K*_n_=R_ab_>*K*_s_>*τ*_m_.

**Table 7:** ANOVA of the central composite design test.

Source	Sum of Squares	df	Squares Mean	F-value	p-value
Model	9.44E+06	14	6.70E+05	23.79	<0.0001**
*K*_n_	2.33E+06	1	2.33E+06	82.84	<0.0001**
*K*_s_	1.83E+05	1	1.83E+05	6.51	0.0254*
τ_m_	1.63E+05	1	1.63E+05	5.79	0.0331*
*R*_ab_	4.41E+06	1	4.41E+06	156.55	<0.0001**
*K*_n_ *K*_s_	8454.02	1	8454.02	0.3004	0.5937
*K*_n_τ_m_	2.88E+05	1	2.88E+05	10.25	0.0076**
*K*_n_ *R*_ab_	7.11E+05	1	7.11E+05	25.28	0.0003**
*K*_s_ *τ*_m_	37413.13	1	37413.13	1.33	0.2713
*K*_s_ *R*_ab_	2.01E+05	1	2.01E+05	7.14	0.0203*
τ_m_ *R*_ab_	12303.19	1	12303.19	0.4372	0.5210
*K_n_* ²	9020.20	1	9020.20	0.3206	0.5817
*K*_s_²	3.77E+05	1	3.77E+05	13.39	0.0033**
*τ*_m_²	10370.33	1	10370.33	0.3685	0.5551
*R*_ab_²	18178.72	1	18178.12	0.6460	0.4372
Residual	3.38E+05	12	28138.71		
Lack of Fit	2.72E+05	10	27155.78	1.05	0.5818
Pure Error	66106.71	2	33053.35		
Cor Total	9.71E+06	26			

*Indicates that the term is significant, P<0.05,** indicates that the termis highly significant,P<0.01; coefficient of determination *R* ^2^ = 0.9652; adjusted determination coefficient*R* ^2^_adj_=0.9247;coefficient of variance CV=16.18%.

Drawing upon the theoretical framework and the analysis shown in **Figures 6C**, **D**, it becomes evident that an increase in *K*_n_ enhances the stiffness of the CKB discrete element model in the normal direction, consequently augmenting *F*_max_. *R*_ab_ is employed to delineate the bonding behavior among particles, as illustrated in **Figure 6D**. It is observed that *F*_max_ escalates with an augmentation of *R*_ab_. A larger bonding radius engenders a stronger bonding force among particles, thereby bolstering the resistance to compression-induced destruction. As shown in **Figure 6E**, *F*_max_ exhibits an initial rise followed by a decline with increasing *K*_s_. Broadly speaking, augmenting *K*_s_ can enhance CKB’s capacity to withstand tangential deformation Equation 2. Nonetheless, owing to the directionality of the compressive force, the tangential stiffness exerts minimal impact on the radial compression of CKB. This finding aligns with the conclusions drawn from the discrete element models of cotton stalks (Zhao et al., 2023) and yam roots (Liu et al., 2022).

Design-Expert 13 software is used to optimize the regression model, with the average CKB compression force (1553.51 N) set as the target. Equation 13 defines the optimization constraints.


(13) $$\left\{ \begin{array}{l} \alpha \to 1553\mathrm{.51}\\ \text{s}\mathrm{.t.}\{\begin{array}{c} 1\times 10^{10}\leq K_{n}\leq 5\times 10^{10}\\ 1\times 10^{10}\leq K_{s}\leq 5\times 10^{10}\\ 1\times 10^{7}\leq \tau_{m}\leq 1\times 10^{10}\\ 0.6\leq R_{ab}\leq 1 \end{array} \end{array}\right.$$

By employing the average value of the actual compression force, the regression equation is solved to ascertain the optimal combination of four factors that align with the compression characteristics of CKB: *K*_n_=3.05×10^10^ N·m^-3^, *K*_s_=3.25×10^10^ N·m^-3^, *σ*_m_= 7.06×10^9^ Pa, *R*_ab_=0.80 mm.

#### 3.2.4 Inversion of significance parameters in GA-BP-GA

**Figure 7:**
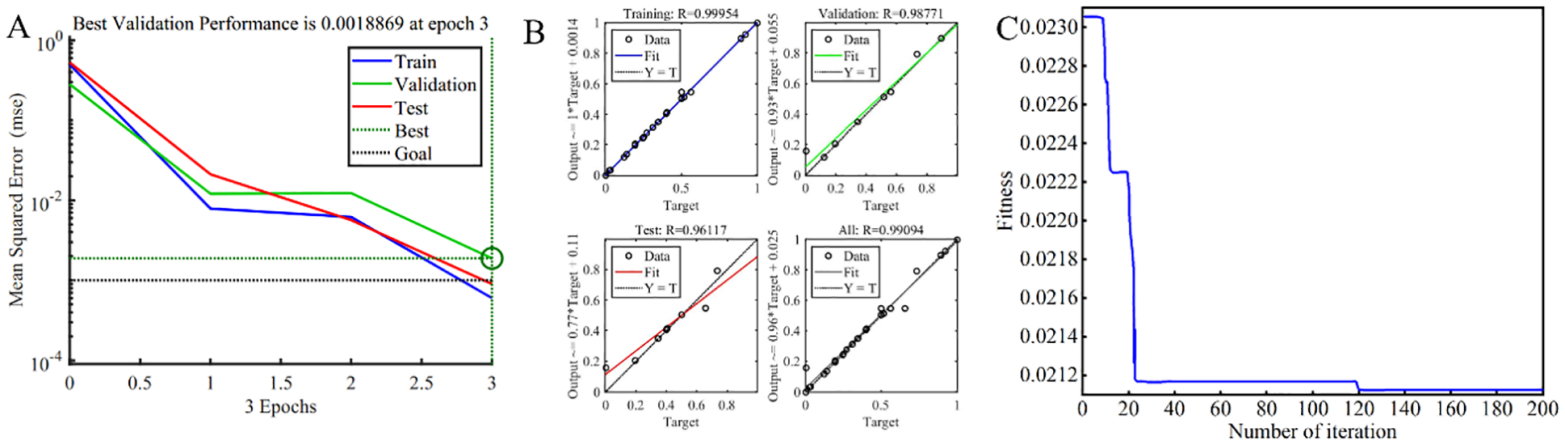
GA-BP-GA evaluation. **(A)** Performance; **(B)** Regression analysis; **(C)** Fitness curve in evolution process.

Using the trial-and-error method, the number of neurons in the hidden layer was explored, ranging from 3 to 13. Due to the limited size of the training dataset, regression fitting may introduce errors. Therefore, the training process was iterated 5 times to determine the optimal number of neurons, which was found to be 13. The performance across the training, validation, and testing datasets for various epochs is shown in **Figure 7A**. The mean squared error (MSE) of the yield reached its minimum at epoch 3, with a value of 0.0018869, indicating the completion of neural network training. The training of the GA-BP network demonstrates rapid convergence and high stability, thereby better meeting the experimental requirements.

Building on the aforementioned optimization, a neural network model with exceptional performance was developed. As shown in **Figure 7B**, post-training analysis reveals correlation coefficients (*R*) for the training, validation, testing, and overall datasets, which are 0.99954, 0.98771, 0.96117, and 0.99094 respectively. These values signify a strong correlation between the predicted and actual data, rendering this neural network model suitable for subsequent experimental analyses.

**Figure 7C** depicts the fitness change curve of random evolutionary generation variations. Initially, GA leverages its group search characteristics, inducing a sharp drop in the fitness of the selected individual. Subsequently, through multiple crossover and selection processes, GA induces minor positive changes in the fitness of the selected individual, gradually approaching the target value. By the 119th generation, the fitness curve gradually converges near 0, indicating minimal disparity between the predicted and target values. After numerous iterations, when the number of evolutionary iterations reaches the target value of 200, GA ceases selection and identifies the individual with the closest fitness. Employing the compression average (1553.51) as the optimization target, the optimal parameters obtained are as follows: *K*_n_=3.67×10^10^ N·m^-3^, *K*_s_=3.42×10^10^ N·m^-3^, *σ*_m_=6.57×10^8^ Pa, *R*_ab_ =0.78 mm.

**Figure 8:**
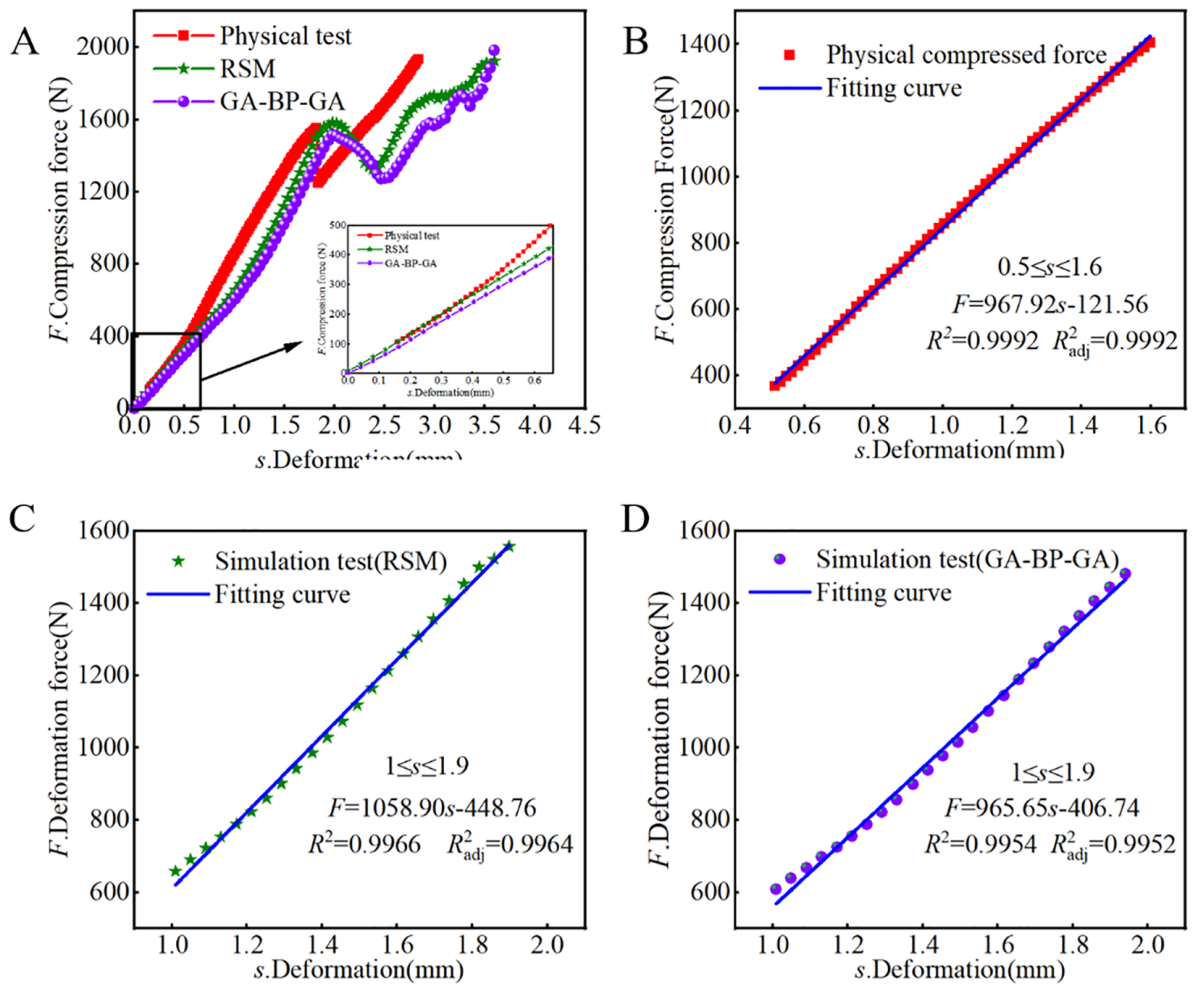
Verification of F-s curves and equation fitting for the elastic deformation stage. **(A)** F-s curves comparison; **(B)** F-s curve fitting in physical test; **(C)** F-s curve fitting in simulation test (RSM); **(D)** F-s curve fitting in simulation test (GA-BP-GA).

**Table 8** presents the parameters obtained through RSM and GA-BP-GA for simulation, with corresponding errors from the actual values being 6.06% and 2.79%, respectively. Notably, the parameters optimized by GA-BP-GA show greater accuracy and closer alignment with the actual values. Analyzing the numerical values of the breaking points, it is observed that the simulated values exceed the actual values, possibly due to the oversimplification of CKB and the neglect of mechanical differences between the epidermis and xylem. Consequently, an actual test curve, close to the previous target value, is selected for curve fitting in the elastic deformation stage. Simultaneously, compression force-displacement curves obtained using two bonding parameters are simulated, and these curves are fitted for the elastic deformation stage. **Figure 8** illustrates similar trends among the three curves, with fitting equation values approaching 1, indicating greater accuracy of the compression force fitting equation in the elastic stage. Notably, the errors in the slopes between the simulated and actual curves are 9.40% and 0.24%, respectively, underscoring the ability of the established CKB discrete element flexibility model to capture force changes during compression.

**Table 8:** Comparative analysis between simulated and actual values.

	Rupture pressure value (N)		The slope of the fitted curve	
Simulated values /N	RSM	GA-BP-GA	RSM	GA-BP-GA
	1459.35	1596.78	1058.90	965.65
Actual values/N	1553.51		967.92	
Error/%	6.06	2.79	9.40	0.24

An important observation is that the simulated deformation upon reaching fracture exceeds the actual value. Specifically, the actual compression curve of CKB fractures at 1.71 mm, followed by a linear increase in compression force. In contrast, the simulation curves fracture at approximately 2.34 mm and 1.98 mm of deformation, respectively. Following the fracture, there is an initial phase of linear growth, followed by force fluctuations attributed to bond breakage between particles.

### 3.3 Verify test results

**Figure 9:**
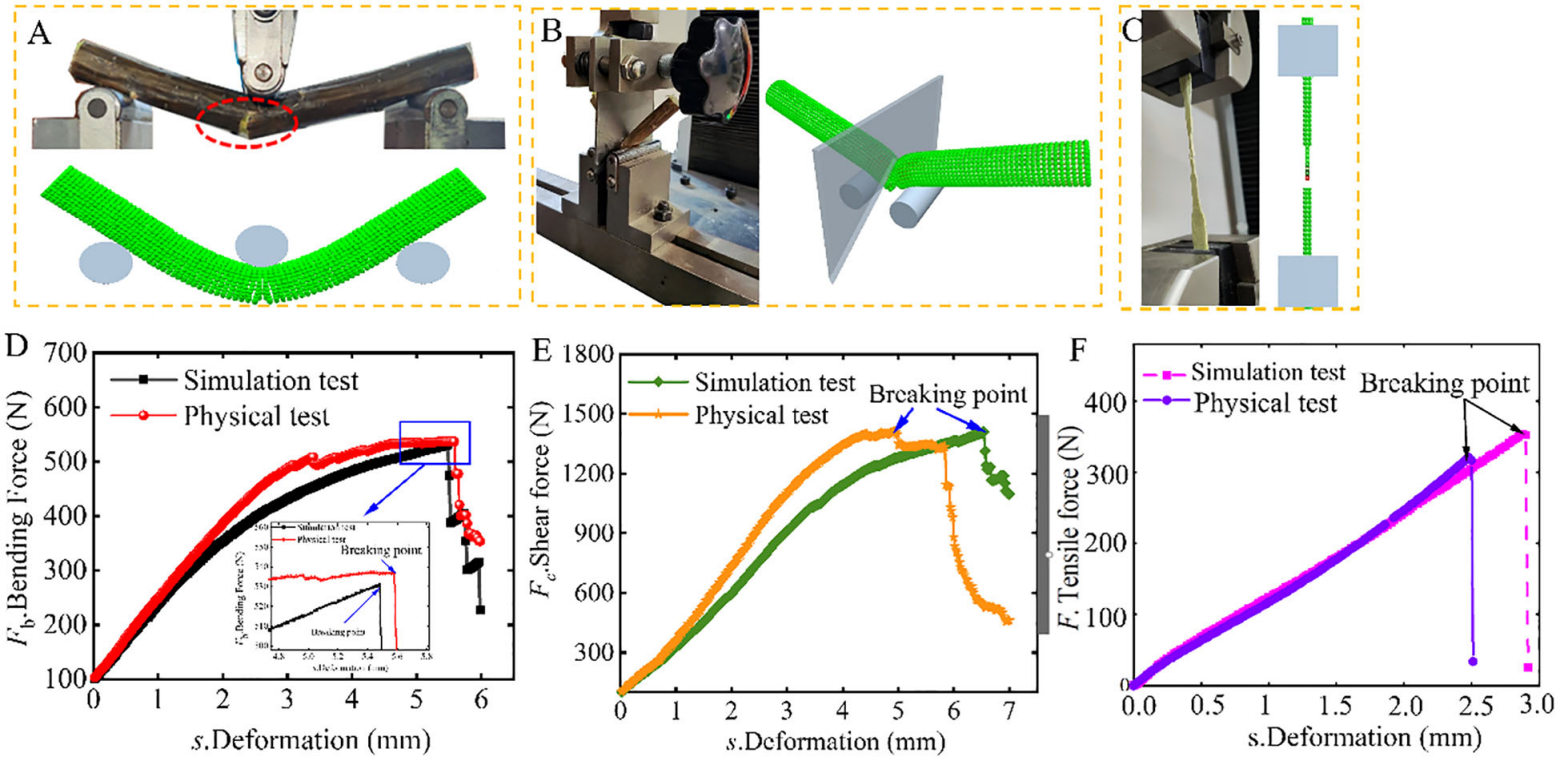
Multi mechanical experimental verification. **(A)** Comparison of three-point bending tests; **(B)** Comparison of double support cutting tests; **(C)** Comparison between tensile simulation test and actual test; **(D)** Comparison of bending force; **(E)** Shear force comparison; **(F)** Comparison of tensile force.

In the CKB simulated bending test, the *F*_wi_ is 544.85 N, while the average *F*_wa_ of the physical bending test is 520.48 N, resulting in a relative error of 4.68%. **Figure 9A** presents a comparison between the simulated bending curve and the actual bending curve, illustrating distinct stages of the CKB fracture process: elastic, elastoplastic, and fully plastic regimes, characterized by a gradual reduction of resistance (**Figure 9D**). Notably, the curves in the elastic stage almost overlap; however, as deformation increases, the slopes of the actual bending curves surpass those of the simulated bending curves, attributed to the simplification of CKB. Upon exceeding the breaking point of CKB, although the load-displacement curve undergoes sharp changes, it still maintains a certain bearing capacity. Furthermore, the sustained tension of fibers on the bottom side of the CKB inhibits further fracture during the actual bending test. With increasing bending force and propagation of microcracks, absorbed energy is released in the form of elastic waves after several repeated failure processes, culminating in the complete fracture of the stem sample. The CKB flexible discrete element model effectively captures the gradual destruction and failure of CKB through the fracture of bonding relationships between particles.

**Figure 9B** shows a comparison between actual experiments and simulation experiments. The mean *F*_c_ of the actual shear test for CKB is 1352.85 N, while the *F*_c_ of the simulated bending test is 1408.80 N, resulting in a relative error of 4.14%. **Figure 9E** shows the shear force-deformation curve, where in the shear force gradually increases as deformation escalates, displaying a trend of initially increasing and then decreasing rate of increase. This pattern mirrors that of the bending curve, albeit requiring a longer duration to reach the maximum crushing point. Upon surpassing the shear strength of the sample, the sample fractures, with force changes exhibiting smoother trends compared to the compression curve, devoid of significant fluctuations. Comparative analysis reveals a similar trend in the relationship between shear force and displacement obtained from simulation to real results, underscoring the capability of the CKB discrete element flexibility model to depict the crushing relationship during shear processes.

To further validate the accuracy of the discrete element model, a tensile test was conducted. **Figure 9C** shown a comparison of the tensile deformation between the simulation and the actual test. During the tensile test, the branches underwent stretching, followed by a local fracture at the center and a complete cross-sectional fracture. The fracture behavior in the simulation model closely mirrors that of the actual branches. **Figure 9F** depicts the variation in tensile force as the branches were stretched. The tensile force changes follow a similar pattern, indicating that the discrete element model replicates the tensile properties of real tea branches. The model’s maximum tensile force is 352.08 N, with an 8.64% relative error compared to the actual test value of 321.63 N. Therefore, the model accurately reflects the tensile properties of CKB.

### 3.4 Discussion

This investigation reveals that CKB possesses distinct mechanical characteristics, markedly differing from those of cotton straw (Jha et al., 2008) and maize straw (Okyere et al., 2022) in stress levels and deformation rates. Intriguingly, CKB displays notable plasticity under compression, with its mechanical attributes heavily contingent upon the orientation of wood fibers. When longitudinally arranged fibrous bundle-like organic materials within the stem undergo pressure against transverse grains, cell walls flatten, resulting in the densification of CKB. Unlike many straw materials, CKB’s cross-grain compression does not lead to unloading failure once it transitions into plasticity fully. Instead, after a certain strain threshold is reached, pressure resistance escalates rapidly.

CKB exhibits an irregular shape, uneven material distribution, and significant variation in mechanical properties, which are further influenced by moisture content and cross-sectional area disparities (Krenke et al., 2018; Nazari Galedar et al., 2008). This investigation constructs a CKB discrete element flexible model with elastic-plastic fracture characteristics, employing a stem sample with a representative moisture content of 32.45% and a diameter of 9.5 mm. In contrast to prior studies (Shi et al., 2022), this paper incorporates a comparison of force change curves under bending and shear failure, enhancing the model’s authenticity. Bonding parameters notably impact the destructive potential of CKB, consistent with findings from prior research (Zhang et al., 2023). Leveraging CKB’s flexibility, the discrete element model enhances *R*_ab_. Interestingly, parameters affecting maximum compression force-*K*_n_, *K*_s_, and *τ*_m_-differ from prior research, potentially due to varying modeling methodologies. This study employs a discrete element flexible model with meta-particles, utilizing X, Y, and Z coordinates to position and rotate particles, minimizing overlap and gaps, thereby reducing setup time and energy. Utilizing a BPNN based on genetic algorithms for optimal bonding parameter determination mitigates the influence of RSM, prone to local optima, a technique frequently employed in chemistry and food sciences (Karambeigi et al., 2023). Drawing from Ding et al. (2023), this study introduces this approach into discrete element simulation parameter optimization, enhancing bonding parameter accuracy.

This study simplifies the branch material as a homogeneous isotropic material, without distinguishing the mechanical properties of the epidermis and xylem. However, the epidermis of agricultural and forestry branches is harder and more brittle, while the xylem exhibits ductility and elastic-plastic behavior. These differences may result in inconsistencies in the simulation’s mechanical parameters. The isotropic assumption overlooks the differences in the mechanical properties of fibers along the longitudinal and radial directions, potentially leading to inaccuracies in stiffness and strength estimation. To address the limitations of the homogeneous isotropic model, future work will use a transverse anisotropic model to more accurately represent the mechanical properties of branches. Experimental calibration of parameters such as elastic modulus, shear modulus, and Poisson’s ratio in various directions will also be conducted. Additionally, to account for the differences between the epidermis and xylem, a layered composite material model will be employed to assign distinct mechanical parameters to each component.

Furthermore, previous studies by the research team have indicated that within a specific moisture content range, the maximum crushing force of CKB decreases with increasing moisture content. While studies have employed specific bonding parameters as variables to delineate the mechanical evolution characteristics of different CKB’s, their accuracy requires further validation. Moving forward, comprehensive consideration of various factors is imperative to establish a robust correlation between the mechanical properties of CKB and the discrete element flexibility model, facilitating enhanced exploration of CKB’s biomechanical properties and expediting its utilization as feedstock.

## 4 Conclusion

This paper proposes a method for calibrating parameters related to the multi-mechanical properties of elasto-plastic materials, specifically focusing on CKB. It conducts comprehensive investigations into the crushing evolution principles of CKB via multi-mechanical tests and integrates these findings with empirical evidence to formulate a discrete CKB model characterized by elasto-plastic crushing properties, termed the metaflexible model.

1. A force-displacement curve for CKB during the stubble period was obtained through a series of diverse mechanical tests. The analysis revealed that the elastic-plastic crushing mechanical behavior of CKB three stages: elastic, elastic-plastic, and fully plastic. CKB exhibited maximum destructive forces of 312.32 N, 1553.51 N, 520.48 N, and 1376.52 N under various loading conditions including tension, compression, bending, and shearing, respectively. Notably, the tensile and bending tests necessitate lower levels of destructive force. The measured elastic modulus, Poisson’s ratio and shear modulus of CKB are 1.74×10^9^ Pa, 0.36 and 6.4×10^8^ Pa respectively.
2. The CKB discrete element flexibility model was constructed utilizing mesh division and the rapid filling method for element particles, augmented by integration with the Bond V2 model. Analysis and calibration via PB tests and central composite design tests respectively led to the establishment of regression equations for normal stiffness per unit area, shear stiffness per unit area, critical tangential stress, bonding radius, and Fmax. The impact of each factor on Fmax was assessed through variance analysis. The findings indicate that the normal stiffness per unit area and bonding radius exert a highly significant influence on Fmax, while the shear stiffness per unit area and critical tangential stress also contribute significantly to Fmax. Utilizing the peak compression crushing force and force-displacement evolution curve as response variables, the parameters of the discrete element flexibility model for Caragana stalks were optimized. The optimal parameters for the bonding model were determined as follows: *K*_n_=3.67×10^10^ N·m^-3^, *K*_s_=3.42×10^10^ N·m^-3^, *σ*_m_=6.57×10^8^ Pa, *R*_ab_=0.78 mm. Simulation tests conducted using bonding parameters yielded an error of 2.79% compared to actual destructive forces, with a slope error of the elastic stage curve at 0.24%.
3. Leveraging the calibration outcomes, a discrete element flexibility model for CKB was formulated. Subsequently, the bending and shear processes from both actual and simulated tests were scrutinized, followed by a comparative analysis of the test outcomes. The discrepancies between the simulated and actual fracture forces in the bending, shear, and tensile tests were 4.68%, 4.14%, and 8.64%, respectively, aligning well with the experimental results. The simulated crushing curve demonstrates a high degree of concordance with the measured curve. This affirms the accuracy of the established CKB discrete element flexibility model, which adeptly delineates the crushing mechanical attributes of CKB. Furthermore, it corroborates the feasibility and efficacy of the modeling approach advocated in this article.

The model will be used to optimize the structural parameters of crushing blades, including their shape, angle, and number, thereby improving crushing efficiency. It will also help analyze the movement trajectory of branches, reduce blockages, and enhance conveyor performance. Additionally, by evaluating energy consumption, it will assist in achieving energy-saving designs and extending the equipment’s lifespan. These future applications will contribute to improving the performance and reliability of crushing machinery, providing significant practical value in engineering.

## Data Availability

The original contributions presented in the study are included in the article/supplementary material. Further inquiries can be directed to the corresponding author.
